# Supplementary material for: Learning curve and surgical outcome of robotic assisted colorectal surgery with ERAS program
Source: Sci Rep. 2022 Nov 29;12:20566. doi: 10.1038/s41598-022-24665-w (PMC9709162; doi:10.1038/s41598-022-24665-w)
Supplement: Supplementary file 1 — Supplementary Information 1. [file 41598_2022_24665_MOESM1_ESM.zip › LSC Raw data-Ñ[▒K/076/H_ñw╝╨░O▒Kñσ.pdf]

正式摘要(電子簽章：000000189268608)		
W52 / W52 ☐ 英文標題(注意:無英文姓名-請補病人基本檔!)	☐【讀取】憑證	

臺中榮民總醫院
出院病歷摘要

轉入號： 轉出號：

[redacted]

(8)流水編號	
(9)入院日期	109/04/12 (2020) 直腸外科 W52 - 161
(10)轉科日期	
(11)出院日期	109/04/18 (2020) 住院天數計 6 日

診斷

- 入院:Distal T-colon cancer
- 出院:Distal T-colon cancer s/p laparoscopic left hemicolectomy on 2020/4/13

主訴

- abdominal pain with fullness for 1 month

病史

- This 39-year-old denied systemic disease.
  According to patient, she suffered from abdominal cramping with fullness for 1 month. She went to LMD first, where colonoscopy revealed D colon cancer, 50cm FAV. Then she went to our hospital. Surgical intervention was suggested. After discussed with patient, he was admitted for scheduled operation.

體檢發現

-
  - General appearance :
    - easy looking , conscious clear , without cardiopulmonary distress
    - Vital signs自動帶入date 體溫(BT) 脈搏(P) 呼吸(R) 血壓(BP) 身高(BH) 體重(BW) BMI
      20200412 36.7℃ 95次/分 19次/分 132/79mmHg 163.1cm 66.3kg 24.9
  - HEENT :
    - Eyes: no anemic conjunctiva, no icteric sclera, normal EOM
    - ENT: no otorrhea, no rhinorrhea, no erythematous mucosa, no injected tonsil, no deformity
  - Neck: supple, freely movable, trachea midline,no deformity, no engorged jugular vein , no palpable thyroid or lymph node enlargement
  - Chest:
    - Inspection: Grossly normal, symmetric and fully expansion, no accessory muscle used, no operation scar, no spider angiomata, no gynecomastia
    - Palpation: no palpable mass or nodule, no subcutaneous emphysema, no tenderness
    - Ausculation: clear breathing sound
  - Heart:
    - Percussion: normal heart size
    - Ausculation: regular heart beat, normal S1, S2, no gallop, no murmur
  - Abdomen:
    - Inspection: in
      - flat contour, symetrically, no scar, no stria, no dilated veins, no rashes
    - Ausculation: normoactive bowel sound, no abdominal bruits
    - palpation: soft, no muscle guarding or rigidity, no local tenderness, no rebounding pain, no palpable mass, no palpable pulsation
    - Percussion: no shifting dulllness, no hepatomegaly, no splenomagaly
-

Peripheral pulsation :	CA	BA	RA	FA	PA	PTA	DPA

Right side :	++	++	++	++	++	++	++/+++
Left side :	++	++	++	++	++	++	++/+++

- Genito-anus: no tenderness or palpable mass lesion or rectal shelf, normal external genitalia, normal anal tone, brown stool

CRS CONDITION

-
    - Lung

手術日期及方法

- 手術日期及方法20200413 1. Laparoscopic left hemicolectomy with side-to-side anastomosis, endoGIA 60/3.8*2, re-inforcement with silk: done.
  2. Vessel ligation: left branch of middle colic and IMV.
  3. Cut margin: proximal 4 cm, distal 20 cm.
  4. Reperitonealization: Nil.
  5. One 12Fr. Penrose drain at left subphrenic region.

住院治療經過

- During admission, laparoscopic left hemicolectomy was arranged on 2020/4/13.
  After operation, we kept post-operative care with pain control, wound care, and tube draingae.
  Her condition improved well with fair appetite, tolerable wound pain and good spirit. Penrose drain was removed on 4/18.
  Base on her stable condition, she was arranged discharge with OPD follow up.

合併症

一般檢查

- 累積報告 :

DATE	TIME	ALB	TP	BIL.T	BIL.D	ALKP	AST	ALT	LDH	CHOL	TG	HDL-C	LDL	UA	NH3	AMY
1090413	0722	3.6	6.7	0.9	0.2	85	21	18	154							
1090414	0859			0.39	0.11		19	18								
1090416	0907	3.1		0.42				10								

DATE	TIME	WBC	RBC	HGB	HCT	MCV	PLT	BLST	NEUT	BAND	SEG	LYM	MON	EOS	BAS
1090414	0902	12330	4.00	7.6			464		73.8			18.7	6.1	1.2	0.2
1090416	0910	8900	4.78	9.7			480		70.7			17.1	5.6	6.3	0.3

DATE	TIME	NA	K	CL	CA	BUN	CREAT	Mg	P	CRP	ESR	PCT	LIPASE	LACTATE
1090413	0722	141	4.0	108	8.4	8	0.69							
1090414	0859	138	3.5		7.7	6	0.69							
1090416	0907	137	4.3		8.3	6	0.65							

特殊檢查

病理報告

- pending

其它

出院時情況

- 3.改門診治療

出院指示

藥名	劑量	劑量單位	用法	頻次	數量	數量單位	現況	天數	單位
Ultracet tab	1	TAB	PO	QID	12	TAB	TH	3	天
Ulstop tab 20mg	1	TAB	PO	BID	6	TAB	TH	3	天

出院計劃

- 門診日期
    - date on 2020/04/21

主治醫師 2062F 蔣鋒帆 / 住院醫師 2327J 王郁棋

正式摘要(電子簽章：**000000189268608)**

W52 / W52

doctorRank:B
